# Supplementary material for: Causes and consequences of sex-chromosome turnovers in Diptera
Source: Evol Lett. 2026 Mar 12;10(3):253–68. doi: 10.1093/evlett/qrag003 (PMC13231274; doi:10.1093/evlett/qrag003)
Supplement: qrag003_Supplemental_Files [file qrag003_supplemental_files.zip › Supplementary_Datasets.zip!/Supplementary_Datasets/Dataset_2_Graphical_representation_outgroups/Drosophilidae_Drosophila_pallidifrons_chromosomes.pdf]

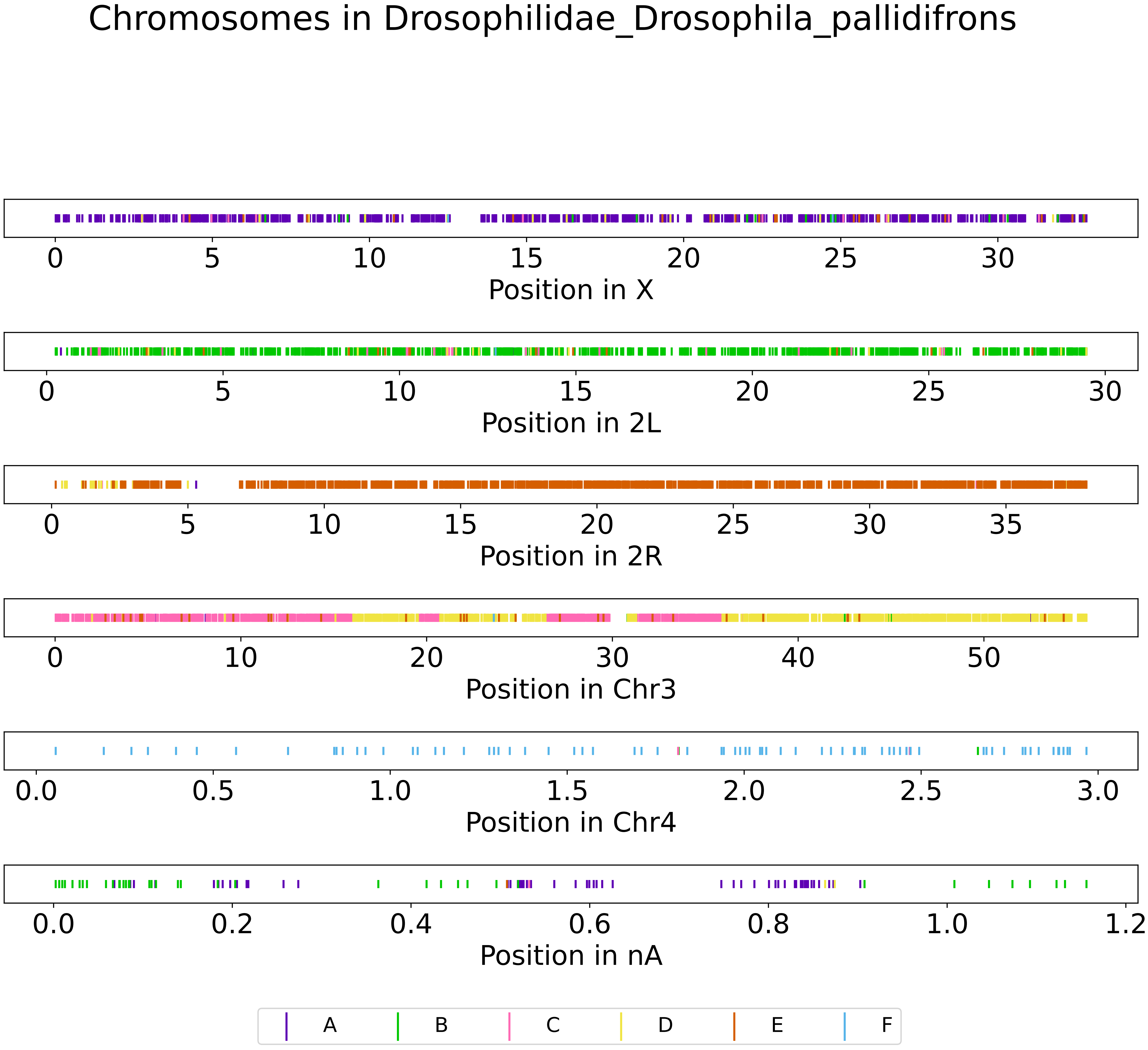
Chromosomes in Drosophilidae_Drosophila_pallidifrons
0
5
10
15
20
25
30
Position in X
0
5
10
15
20
25
30
Position in 2L
0
5
10
15
20
25
30
35
Position in 2R
0
10
20
30
40
50
Position in Chr3
0.0
0.5
1.0
1.5
2.0
2.5
3.0
Position in Chr4
0.0
0.2
0.4
0.6
0.8
1.0
1.2
Position in nA
A
B
C
D
E
F
